# Inositol Depletion Restores Vesicle Transport in Yeast Phospholipid Flippase Mutants

**DOI:** 10.1371/journal.pone.0120108

**Published:** 2015-03-17

**Authors:** Kanako Yamagami, Takaharu Yamamoto, Shota Sakai, Tetsuo Mioka, Takamitsu Sano, Yasuyuki Igarashi, Kazuma Tanaka

**Affiliations:** 1 Division of Molecular Interaction, Institute for Genetic Medicine, Hokkaido University Graduate School of Life Science, Sapporo, Japan; 2 Laboratory of Biomembrane and Biofunctional Chemistry, Graduate School of Advanced Life Science, and Frontier Research Center for Post-Genome Science and Technology, Hokkaido University, Sapporo, Japan; Simon Fraser University, CANADA

## Abstract

In eukaryotic cells, type 4 P-type ATPases function as phospholipid flippases, which translocate phospholipids from the exoplasmic leaflet to the cytoplasmic leaflet of the lipid bilayer. Flippases function in the formation of transport vesicles, but the mechanism remains unknown. Here, we isolate an arrestin-related trafficking adaptor, *ART5*, as a multicopy suppressor of the growth and endocytic recycling defects of flippase mutants in budding yeast. Consistent with a previous report that Art5p downregulates the inositol transporter Itr1p by endocytosis, we found that flippase mutations were also suppressed by the disruption of *ITR1*, as well as by depletion of inositol from the culture medium. Interestingly, inositol depletion suppressed the defects in all five flippase mutants. Inositol depletion also partially restored the formation of secretory vesicles in a flippase mutant. Inositol depletion caused changes in lipid composition, including a decrease in phosphatidylinositol and an increase in phosphatidylserine. A reduction in phosphatidylinositol levels caused by partially depleting the phosphatidylinositol synthase Pis1p also suppressed a flippase mutation. These results suggest that inositol depletion changes the lipid composition of the endosomal/TGN membranes, which results in vesicle formation from these membranes in the absence of flippases.

## Introduction

In eukaryotic cells, phospholipids are asymmetrically distributed across the plasma membrane bilayer; phosphatidylserine (PS) and phosphatidylethanolamine (PE) are enriched in the cytoplasmic leaflet, whereas phosphatidylcholine (PC) and sphingolipids are located in the exoplasmic leaflet. Phospholipid flippases are type 4 P-type ATPases that translocate (‘flip’) phospholipids from the exoplasmic to the cytoplasmic leaflet, thereby generating and maintaining the phospholipid asymmetry of the plasma membrane and organellar membranes [1–4]. In mammals, flippases are involved in spermatogenesis [5], B cell development [6, 7], neuronal growth [8], hippocampus-dependent learning [9], and cell migration [10]. The molecular mechanisms that underlie these important cellular functions remain to be elucidated, but yeast offers a well-characterized model system in which to study the functions of flippases.

The yeast *Saccharomyces cerevisiae* encodes five flippases: Drs2p, Dnf1p, Dnf2p, Dnf3p, and Neo1p. Of these, Drs2p, Dnf3p, and Dnf1p/Dnf2p form complexes with non-catalytic subunits of the Cdc50 family: Cdc50p, Crf1p, and Lem3p, respectively. These interactions are required for the ER exit, proper localization, function, and activity of the flippases [11–15]. Therefore, the defective *drs2*Δ, *dnf1*Δ *dnf2*Δ, and *dnf3*Δ mutants are phenocopied by *cdc50*Δ, *lem3*Δ, and *crf1*Δ mutants, respectively [11, 12].

Phenotypic analyses of yeast flippase mutants suggest that they are involved in vesicle transport pathways. Cdc50p-Drs2p, Lem3p-Dnf1/2p, and Crf1p-Dnf3p are collectively essential for viability and are required for vesicular transport from early endosomes during the endocytic recycling pathway [12]. Cdc50p-Drs2p plays an especially prominent role in this pathway and is also involved in the formation of clathrin-coated vesicles from early endosomal/TGN membranes [16–19]. The AP-1 clathrin adaptor functions downstream of Cdc50p-Drs2p [18, 19], but the underlying mechanisms are unknown.

Neo1p does not associate with a Cdc50 family member [11] and is independently essential for viability [20]. Neo1p is involved in membrane trafficking from the *cis*-Golgi to the ER [21] and within the endosomal/Golgi system [22]. Although the flippase activity of Neo1p has not been demonstrated, Neo1p functions redundantly with Cdc50p-Drs2p in the endocytic recycling pathway [23].

Phospholipid flipping by flippases has been implicated in the formation of membrane curvature, which would assist in vesicle formation [24]. Because lipid composition is an important determinant for the physical property of membranes, it may have a crucial impact on flippase-mediated vesicle formation.

Inositol is an essential precursor in the synthesis of phosphatidylinositol (PI), which in turn, is a precursor of many important signaling molecules [25, 26], including phosphoinositides [27], inositol pyrophosphates [28, 29], sphingolipids [30, 31], and glycosylphosphatidylinositol anchor proteins [32]. PI is synthesized from inositol and cytidine diphosphate diacylglycerol (CDP-DAG) in a reaction catalyzed by Pis1p. Yeast cells are capable of growing in the absence of exogenous inositol, because they can convert D-glucose 6-phosphate to D-myo-inositol 3-phosphate through a reaction catalyzed by Ino1p. However, under inositol-depleted conditions (defined as the absence of inositol from the growth medium), PI levels are decreased, phospholipid synthesis genes are transcriptionally activated, and stress responses (such as the unfolded protein response [UPR]) are induced [33].

In this study, we isolated an arrestin homologue, *ART5*, as a multicopy suppressor of the growth defects of flippase mutants. Consistent with a previous report that Art5p downregulates the inositol transporter Itr1p through endocytosis, the defects of flippase mutants were also suppressed by the *itr1*Δ mutation, as well as by inositol depletion from the culture medium. Inositol depletion efficiently suppressed the growth and membrane trafficking defects in all of the flippase mutants, suggesting that the membranes of inositol-depleted cells do not require flippases for vesicle formation.

## Materials and Methods

### Media and genetic techniques

Unless otherwise specified, strains were grown in rich medium (YPDA: 1% yeast extract [Difco Laboratories, Detroit, MI, USA], 2% bacto-peptone [Difco Laboratories], 2% glucose [Wako Pure Chemical Industries Ltd., Osaka, Japan], and 0.01% adenine [Wako Pure Chemical Industries]). Strains carrying plasmids were selected in synthetic medium (SD: 0.67% yeast nitrogen base without amino acids [Difco Laboratories] and 2% glucose) containing the required nutritional supplements. SDA medium was SD medium containing 0.5% casamino acids (Difco Laboratories). For induction of the *GAL1* promoter, 3% galactose (Wako Pure Chemical Industries) and 0.2% sucrose (Wako Pure Chemical Industries) were used as carbon sources instead of glucose (in the YPGA, SG-Leu, and SGA-Ura mediums). When expression from the *GAL1* promoter was attenuated, 2% raffinose and 0.005% galactose were used as carbon sources. SD medium with or without 10 mg/l inositol was prepared as described [34]. Duramycin, miltefosine, and tunicamycin were from Sigma-Aldrich (St. Louis, MO, USA). Papuamide B and aureobasidin A were from Flintbox, Wellspring Worldwide (Chicago, IL, USA) and Takara Bio Inc. (Shiga, Japan), respectively. Standard genetic manipulations of yeast were performed as described previously [35]. Yeast transformations were performed by the lithium acetate method [36, 37]. *Escherichia coli* strains DH5α and XL1-Blue were used for the construction and amplification of plasmids.

### Strains and plasmids

The yeast strains used in this study are listed in Table 1. Because flippase mutants exhibit defects in tryptophan uptake [38], we constructed a wild-type strain in which *trp1–63* was replaced with *TRP1* (YKT1066) [23], and most strains used in this study were derived from this strain. PCR-based procedures were used to construct gene deletions and gene fusions with the *GAL1* promoter, GFP, and mRFP [39]. Some gene deletions (*itr1*Δ, *itr2*Δ, and *opi1*Δ) were constructed by transformation with PCR products from knockout strains. All constructs produced by the PCR-based procedure were verified by colony PCR amplification to confirm that replacement occurred at the expected locus. When required, the selection markers of the mutant alleles were changed by cassette exchange [40]. The plasmids used in this study are listed in Table 2. *ART5*-*PYm* (P533A/Y535A) and *ART5*-*AMm* (G231A/P236A/F237A) mutations were constructed using the QuikChange II site-directed mutagenesis kit (Agilent Technologies, Santa Clara, CA, USA). YCp-*HAC1* (238 type S238A) and pRS426-*ALY1* and-*ALY2* were kindly provided by Dr. Kazutoshi Mori (Kyoto University) and Dr. Allyson F. O’Donnell (University of Pittsburgh), respectively.

**Table 1:** *S*. *cerevisiae* strains used in this study.

Strain	Genotype		Reference or source
YEF473	*MAT*a/α	*lys2–810/lys2–810 ura3–52/ura3–52 his3*Δ*-200/his3*Δ*-200 trp1*Δ*-63/trp1*Δ*-63 leu2*Δ*-1/leu2*Δ*-1*	[39]
BY4743	*MAT*a /α	*LYS2/lys2*Δ*0 ura3*Δ*0/ura3*Δ*0 his3*Δ*1/his3*Δ*1 leu2*Δ*0/leu2*Δ*0 met15Δ0/MET15*	[41]
YKT38	*MAT*a	*lys2–801 ura3–52 his3*Δ*-200 leu2*Δ*-1 trp1*Δ*-63*	[42]
YKT1066	*MAT*a	*lys2–801 ura3–52 his3*Δ*-200 leu2*Δ*-1 TRP1*	[23]
YKT1286	*MAT*a	*HphMX4*::*P*_*GAL1*_ *–3HA-CDC50 gcs1*Δ::*KanMX6*	[18]
YKT1649	*MAT*α	*HIS3MX6*::*P*_*GAL1*_ *–3HA-CDC50 neo1–101*	[23]
YKT1511	*MAT*a	*KanMX6*::*P*_*GAL1*_ *–3HA-CDC50 crf1*Δ::*HphMX3 TRP1*	[23]
YKT1529	*MAT*a	*KanMX6*::*P*_*GAL1*_ *–3HA-CDC50 dnf1*Δ::*HIS3MX6 crf1*Δ::*HphMX3 TRP1*	[23]
YKT1513	*MAT*a	*KanMX6*::*P*_*GAL1*_ *–3HA-CDC50 lem3*Δ::*HIS3MX6 crf1Δ*::*HphMX3 TRP1*	[23]
YKT1932	*MAT*α	*KanMX6*::*P*_*GAL1*_ *–3HA-NEO1 TRP1*	This study
YKT1933	*MAT*α	*KanMX6*::*P*_*GAL1*_ *–3HA-CDC50 crf1*Δ::*HphMX3 URA3*::*P*_*TPI1*_ *-GFP-SNC1 TRP1*	This study
YKT1910	*MAT*a	*KanMX6*::*P*_*GAL1*_ *–3HA-NEO1 LEU2*:: *P*_*TPI1*_ *-mRFP-SNC1 TRP1*	This study
YKT715	*MAT*a	*lem3*Δ::*TRP1*	[11]
YKT1866	*MAT*α	*itr1*Δ::*KanMX4 itr2* Δ::*HphMX4 TRP1*	This study
YKT1934	*MAT*a	*TRP1*::*P*_*GAL1*_ *–3HA-CDC50 dnf1* Δ::*HIS3MX6 crf1* Δ::*HphMX3 itr1* Δ::*KanMX4*	This study
YKT1935	*MAT*a	*TRP1*::*P*_*GAL1*_ *–3HA-CDC50 dnf1* Δ:: *HIS3MX6 crf1* Δ::*HphMX3 itr1* Δ::*KanMX4 itr2* Δ::*NatMX4*	This study
YKT1914	*MAT*α	*HIS3MX6*::*P*_*GAL1*_ *–3HA-CDC50 lem3* Δ::*TRP1 crf1* Δ::*HphMX3 itr1* Δ::*KanMX4 itr2* Δ::*NatMX4*	This study
YKT1915	*MAT*α	*HIS3MX6*::*P*_*GAL1*_ *–3HA-CDC50 lem3* Δ::*TRP1 crf1* Δ::*HphMX3 itr1* Δ::*KanMX4*	This study
YKT1877	*MAT*a	*KanMX6*::*P*_*GAL1*_ *–3HA-NEO1 itr1* Δ::*HIS3MX6 TRP1*	This study
YKT1881	*MAT*a	*KanMX6*::*P*_*GAL1*_ *–3HA-NEO1 itr1* Δ::*HIS3MX6 itr2* Δ::*NatMX4 TRP1*	This study
YKT1909	*MAT*a	*KanMX6*::*P*_*GAL1*_ *–3HA-NEO1 HIS3MX6*::*P*_*GAL1*_ *–3HA-CDC50 TRP1*	This study
YKT1887	*MAT*a	*KanMX6*::*P*_*GAL1*_ *–3HA-NEO1 HIS3MX6*::*P*_*GAL1*_ *–3HA-CDC50 lem3* Δ::*TRP1 crf1* Δ::*HphMX3*	This study
YKT1944	*MAT*a	*cdc50* Δ::*HIS3MX6*	This study
YKT1945	*MAT*a	*cdc50* Δ:: *HIS3MX6 dnf1* Δ::*KanMX4 TRP1*	This study
YKT1912	*MAT*a	*LEU2*::*P*_*TPI1*_ *-mRFP-SNC1 TRP1*	This study
YKT1936	*MAT*α	*KanMX6*::*P*_*GAL1*_ *–3HA-CDC50 dnf1*Δ::*HIS3MX6 crf1Δ*::*HphMX3 LEU2*::*P*_*TPI1*_ *-mRFP-SNC1 TRP1*	This study
YKT1937	*MAT*α	*KanMX6*::*P*_*GAL1*_ *–3HA-CDC50 lem3*Δ::*HIS3MX6 crf1Δ*::*HphMX3 LEU2*::*P*_*TPI1*_ *-mRFP-SNC1 TRP1*	This study
YKT1120	*MAT*a	*HIS3MX6*::*P*_*GAL1*_ *–3HA-CDC50 lem3*Δ::*TRP1 crf1*Δ::*HphMX4*	This study
YKT1660	*MAT*a	*KanMX6*::*P*_*GAL1*_ *–3HA-NEO1*	[23]
YKT1844	*MAT*a	*sec6–4 URA3*::*P*_*GPD*_ *-mRFP1-Lact-C2*	[43]
YKT1854	*MAT*a	*sec6–4 HIS3MX6*::*P*_*GAL1*_ *–3HA-CDC50 lem3* Δ::*TRP1 crf1* Δ::*HphMX4 URA3*::*P*_*GPD*_ *-mRFP1-Lact-C2*	[43]
YKT1938	*MAT*a	*opi1* Δ::*KanMX4*	This study
YKT1510	*MAT*a	*KanMX6*::*P*_*GAL1*_ *–3HA-CDC50 crf1* Δ::*HphMX3*	This study
YKT1939	*MAT*a	*HIS3MX6*::*P*_*GAL1*_ *–3HA-CDC50 crf1* Δ::*HphMX3 opi1* Δ::*KanMX4*	This study
YKT1940	*MAT*α	*HIS3MX6*::*P*_*GAL1*_ *–3HA-CDC50 lem3* Δ::*TRP1 crf1* Δ::*HphMX3 opi1* Δ::*KanMX4*	This study
YKT1941	*MAT*a	*KanMX6*::*P*_*GAL1*_ *–3HA-NEO1 opi1* Δ::*HphMX4*	This study
YKT1697	*MAT*a	*cdc50* Δ::*TRP1*	This study
YKT1942	*MAT*a	*KanMX6*::*P*_*GAL1*_ *–3HA-PIS1*	This study
YKT1943	*MAT*a	*KanMX6*::*P*_*GAL1*_ *–3HA-PIS1 cdc50* Δ::*TRP1*	This study
KKT116	*MAT*a	*HphMX4*::*P*_*GAL1*_ *–3HA-CDC50 fpk1* Δ::*KanMX6*	This study
KKT466	*MAT*a	*hac1* Δ::*KanMX4*	This study

YKT and KKT strains are isogenic derivatives of YEF473 and BY4743, respectively.

Only relevant genotypes are described.

**Table 2:** Plasmids used in this study.

Plasmid	Characteristics	Reference or source
YCplac111	*LEU2 CEN*	[39]
YEplac195	*URA3* 2μm	[44]
YEplac181	*LEU2* 2μm	[44]
pKT1263 [YEplac195-CDC50]	*CDC50 URA3* 2μm	[23]
pKT1720 [YEplac195-ART5]	*ART5 URA3* 2μm	This study
pKT1469 [YEplac195-NEO1]	*NEO1 URA3* 2μm	[23]
pKT2135 [YEplac195-RIM8]	*RIM8 URA3* 2μm	This study
pKT2136 [YEplac195-ROD1]	*ROD1 URA3* 2μm	This study
pKT2137 [YEplac195-ROG3]	*ROG3 URA3* 2μm	This study
pKT1881 [pRS426-ALY1]	*ALY1 URA3* 2μm	[45]
pKT1882 [pRS426-ALY2]	*ALY2 URA3* 2μm	[45]
pKT2088 [YEplac195-ART5-PYm (P533A/Y535A)]	*art5* (*P533A*/*Y535A*) *URA3* 2μm	This study
pKT2138 [YEplac195-ART5-AMm (G231A/P236A/F237A)]	*art5* (*G231A*/*P236A*/*F237A*) *URA3* 2μm	This study
pKT1444 [pRS416-GFP-SNC1 pm]	*P*_*TPI1*_-*GFP-SNC1* (*pm*) *URA3 CEN*	[46]
pKT1491 [pRS315-GFP-SNC1 pm]	*P*_*TPI1*_ *-GFP-SNC1 (pm) LEU2 CEN*	[43]
pKT2139 [YCp-HAC1 (238 type S238A)]	*HAC1* (*S238A*) *LEU2 CEN*	[47]
pKT1259 [YEplac181-CDC50]	*CDC50 LEU2* 2μm	[42]
pKT1788 [pRS425-NEO1]	*NEO1 LEU2* 2μm	[23]
pKT1719 [YEplac181-ART5]	*ART5 LEU2* 2μm	This study
pKT1754 [YEplac181-CHO1]	*CHO1 LEU2* 2μm	This study

### Isolation of *ART5* as a multicopy suppressor of *P*_*GAL1*_ *-CDC50 gcs1* Δ and *P*_*GAL1*_ *-CDC50 fpk1* Δ mutations

We previously isolated *ART5* as a multicopy suppressor of the *P*_*GAL1*_ *-CDC50 neo1–101* mutations [23], but did not characterize it further. *ART5* was also isolated in two independent screenings as described below. The *P*_*GAL1*_ *-CDC50 gcs1* Δ strain (YKT1286) was transformed with a yeast genomic library inserted into the multicopy plasmid YEp13 [48]. Transformants were grown on SG-Leu agar plates at 30°C for recovery, replica-plated on SD-Leu plates, and incubated for 2–3 days at 30°C. From transformants that grew in the Cdc50p-depleted condition, plasmids were recovered and re-introduced into YKT1286. Of the seven plasmids that demonstrated clear suppression, four plasmids contained *CDC50*, as determined by DNA sequencing. Of the remainder, two encoded the N-terminal half of *SWH1* and one encoded *ART5*, as determined by subcloning analysis.

The *P*_*GAL1*_ *-CDC50 fpk1*Δ strain (KKT116) was transformed with a yeast genomic library inserted into the multicopy plasmid YEp24 [49]. Transformants were grown on SGA-Ura plates at 30°C for recovery, replica-plated on SD-Ura plates, and incubated at 30°C for 2–3 days. We selected 200 transformants that reproducibly grew on the glucose-containing plates. Of these, we eliminated those that grew at 18°C, because they were likely to contain a plasmid harboring *CDC50*. Transformants with *FPK1* were identified by colony PCR and were also eliminated. From the remaining transformants, plasmids were recovered and grouped by restriction enzyme mapping. Plasmids that conferred suppression after retransformation belonged to 4 classes, identified by DNA sequencing: those that contained *ART5*, *KIN82* (*FPK2*), *YCK1*, or *YCK2*.

### Microscopic observations

Cells were observed using a Nikon Eclipse E800 microscope (Nikon Instec, Tokyo, Japan) equipped with an HB-10103AF super high-pressure mercury lamp and a 1.4 numerical aperture, 100× Plan Apo oil immersion objective lens with appropriate fluorescence filter sets or with differential interference contrast microscopy. Images were acquired using a cooled charge-coupled device digital camera (C4742–95–12NR; Hamamatsu Photonics, Hamamatsu, Japan) using the AQUACOS-MOS software (Hamamatsu Photonics). To visualize GFP- or mRFP-tagged proteins, cells were grown under the indicated conditions, harvested, and resuspended in SDA- or SD-based medium. Cells were mounted onto microslides and immediately observed using a GFP band-pass or G-2A (for mRFP) filter set.

### Quantitation of phospholipids using liquid chromatography mass spectrometry

Cells were grown in 50 ml of the appropriate medium to an optical density (600 nm) of 0.5–0.8 for 12–16 h at 30°C. Phospholipids were extracted according to the method of Bligh and Dyer [50]. Briefly, the cells (30 OD_600_ units) were harvested by centrifugation, suspended in 800 μl of 0.9% KCl, and transferred to a screw-cap glass tube. After the addition of 2 ml of methanol, 1 ml of chloroform, 100 μl of 1 N HCl in methanol, and internal lipid standards, the mixture was vortexed with glass beads for 1 min to disrupt the cell wall. After incubation at 37°C for 2 h, 1 ml each of 0.9% KCl and chloroform was added, and the samples were mixed. The lipid-containing lower phase was recovered by centrifugation and dried under nitrogen gas. The internal standards used were 10 μl of 100 μM solutions of PC (17:0/17:0), PE (17:0/17:0), and PI (8:0/8:0), or 10 μl of a 50 μM solution of PS (17:0/17:0) (Avanti Polar Lipids, Alabaster, AL, USA). The internal standards’ exact lipid concentrations were determined using phosphorus analysis.

A Prominence UFLC system (Shimadzu, Kyoto, Japan) coupled to a TripleTOF 5600 System (AB SCIEX, Foster City, CA, USA) was used to quantitate the phospholipids. Extracted samples were injected on a Luna Silica column (3 μm, 2.0 × 150 mm, Phenomenex, CA, USA) at a flow rate of 0.2 ml/min, and the column temperature was kept at 25°C. Solvent A (chroloform:methanol:2-propanol:ammonia water [80:12.5:7:0.5, v/v/v/v] containing 5 mM ammonium formate) and solvent B (methanol:2-propanol:ammonia water [92.5:7:0.5, v/v/v] containing 5 mM ammonium formate) were used as eluents. The samples were eluted through the following gradient conditions: solvent A for 0.5 min, followed by a linear gradient to a solvent A/solvent B mixture (30:70, v/v) over 30 min. After 10 min at 70% solvent B, the gradient was changed to solvent A over 5 min and maintained for 10 min to equilibrate the column. The optimal conditions for the ionization of each phospholipid were determined. TOF-MS analysis was run in the positive ion mode for each molecular species of PC, PE, and PS, and negative ion mode for PI with the following instrument parameters: curtain gas of 10 psi, ion spray voltage of 5500 in positive ion mode (-4500 in negative ion mode), and temperature of 300°C. The data-independent MS/MS scan mode was used to determine the molecular species of each phospholipid. The amount of each phospholipid molecular species detected by TOF-MS analysis was calculated by relating the peak area of each species to the peak area of the corresponding internal standard and the standard curve of each internal standard. The total amount of each phospholipid was calculated by the sum of the amount of each molecular species. Data acquisition and analysis were performed using Analyst Software version 1.4.1 (AB SCIEX).

### Isolation of secretory vesicles from the flippase mutant

Secretory vesicles were isolated from a flippase mutant carrying the *sec6–4* mutation as described previously [43]. Briefly, cells were grown at 30°C for 12 h to early- to mid-logarithmic phase (OD_600_ of 0.5–0.7) in 0.5 L of SD medium with or without inositol, followed by further incubation at 37°C for 2 h to allow the accumulation of secretory vesicles. The membrane fraction containing the secretory vesicles was obtained using subcellular fractionation and was further subjected to Nycodenz gradient fractionation. To quantitatively estimate the amount of isolated secretory vesicles in each fraction, the fluorescence intensity of mRFP-Lact-C2, which binds to PS on the surface of secretory vesicles, were determined and total phospholipid phosphates were measured.

## Results

### Identification of *ART5* as a multicopy suppressor of flippase mutations

**Fig 1:**
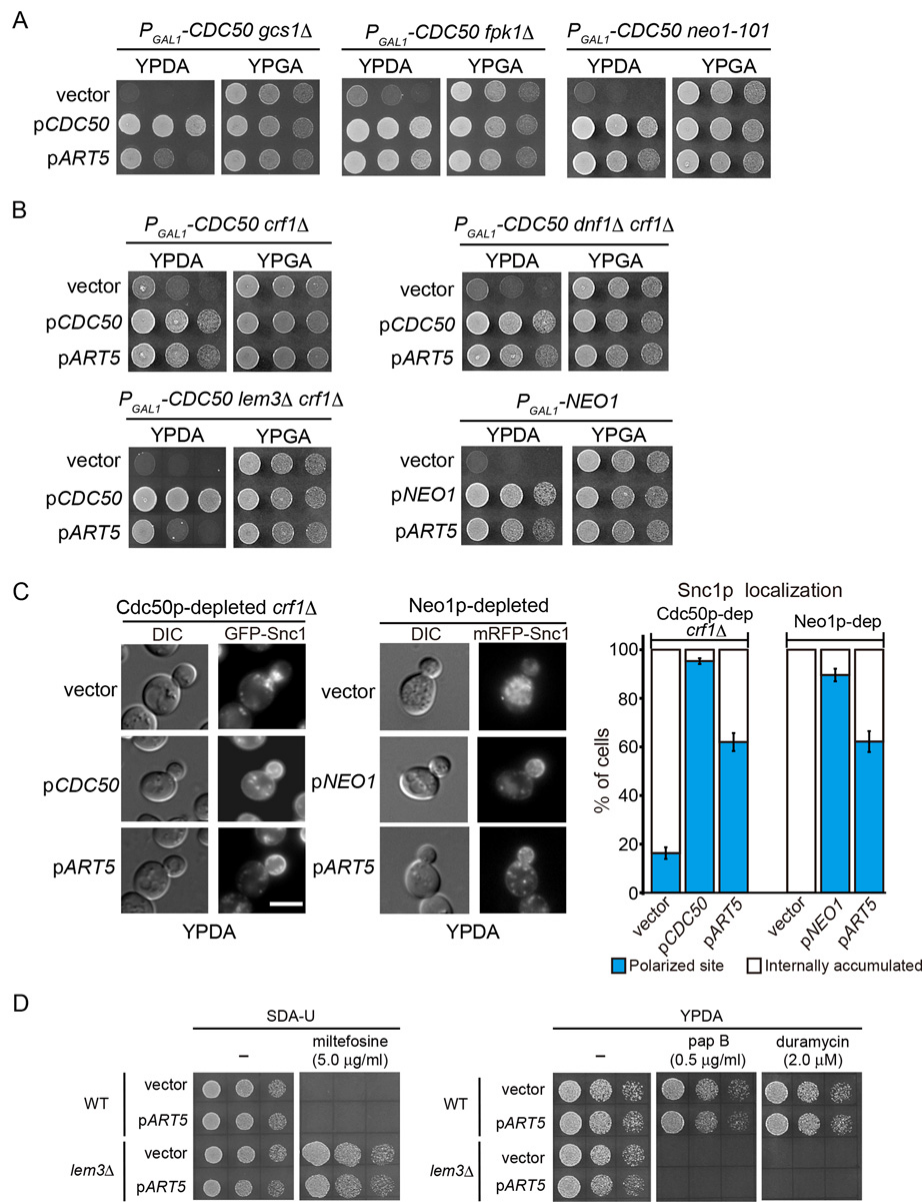
Identification of *ART5* as a multicopy suppressor of flippase mutations. (A) Suppression of growth defects by overexpressing *ART5* in Cdc50p-depleted mutants carrying a mutation synthetically lethal with *cdc50*Δ. Cells were grown to early log phase in SGA-Ura medium, washed, and adjusted to a concentration of 2.5 × 10^7^ cells/ml. Drops of 4 μl from 5-fold serial dilutions were spotted onto a YPDA (Cdc50p-depleted) or YPGA (Cdc50p-expressed) agar plate, followed by incubation at 30°C for 1 day. The strains used were YKT1286 (*P*_*GAL1*_ *-CDC50 gcs1*Δ), KKT116 (*P*_*GAL1*_ *-CDC50 fpk1*Δ), and YKT1649 (*P*_*GAL1*_ *-CDC50 neo1–101*), all carrying YEplac195 (vector), pKT1263 (p*CDC50*), or pKT1720 (p*ART5*). (B) Suppression of growth defects in flippase mutants by overexpression of *ART5*. Cells were grown and examined as in (A), except that the *P*_*GAL1*_ *-CDC50 crf1*Δ mutant was incubated at 25°C for 2 days. The strains used were YKT1511 (*P*_*GAL1*_ *-CDC50 crf1*Δ), YKT1529 (*P*_*GAL1*_ *-CDC50 dnf1*Δ *crf1*Δ), YKT1513 (*P*_*GAL1*_ *-CDC50 lem3*Δ *crf1*Δ), and YKT1932 (*P*_*GAL1*_ *-NEO1*), all carrying each of the plasmids in (A), except YKT1932 carried pKT1469 (p*NEO1*) as a positive control. (C) Suppression of the defects in membrane trafficking in flippase mutants by overexpression of *ART5*. Cells were grown in YPDA medium at 25°C for 14 h (Cdc50p-depleted *crf1*Δ) or at 30°C for 12 h (Neo1p-depleted), followed by microscopic observation of small- or middle-budded cells. The percent of cells with polarized GFP- or mRFP-Snc1p was determined (n>100) and is shown with the mean ± standard deviation of three independent experiments. Representative images are shown. The strains used were YKT1933 (*P*_*GAL1*_ *-CDC50 crf1*Δ *GFP-SNC1*) and YKT1910 (*P*_*GAL1*_ *-NEO1 mRFP-SNC1*), both carrying each of the plasmids in (A), except YKT1910 carried pKT1469 (p*NEO1*) as a positive control. Bar: 5 μm. (D) Failure of *ART5* overexpression to suppress the alkylphosphocholine resistance and phospholipid-binding peptide sensitivity in a flippase mutant. Cells were grown to early log phase in SDA-Ura medium, washed, and adjusted to a concentration of 5.0 × 10^6^ cells/ml. Drops of 10 μl and 4 μl from 5-fold serial dilutions were spotted onto SDA-Ura containing 5.0 μg/ml miltefosine and YPDA containing 0.5 μg/ml papuamide B (pap B) or 2.0 μM duramycin agar plates, respectively, followed by incubation at 30°C for 1 day. The strains used were YKT1066 (WT) and YKT715 (*lem3*Δ), both carrying YEplac195 (vector) or pKT1720 (p*ART5*).

We previously identified numerous mutations as synthetically lethal with *cdc50*Δ, including *gcs1*Δ, *fpk1*Δ, and *neo1–101* [18, 23, 51]. Gcs1p is an Arf GTPase-activating protein (Arf-GAP), Fpk1p is an upstream protein kinase that phosphorylates Dnf1p and Dnf2p, and *neo1–101* carries a point mutation in the carboxyl-terminal cytosolic region of Neo1p. To identify genes involved in the regulation or function of Cdc50p-Drs2p, we performed multicopy suppressor screening on double mutants of each of the previously isolated genes, with *CDC50* under the control of the glucose-repressible *GAL1* promoter (this promoter mutation is hereby referred to as “Cdc50-depleted”). *ART5* (*YGR068C*), which encodes a member of the arrestin-related trafficking adaptors (ARTs) protein family [52, 53], was isolated in all screens (Fig. 1A), suggesting that overexpression of *ART5* suppresses *cdc50*Δ defects. Hence, we next examined whether *ART5* overexpression suppressed flippase mutations.

The *cdc50*Δ single mutant grows normally at 30°C, but not at 25°C or below [42]. Overexpression of *ART*5 suppressed the growth defects of the Cdc50p-depleted *crf1*Δ mutant at 25°C (Fig. 1B). The Cdc50p-depleted *dnf1*Δ *crf1*Δ and *lem3*Δ *crf1*Δ mutants exhibited severe growth defects and lethality, respectively, which were suppressed by overexpression of *ART5*. We found that *ART5* overexpression even suppressed the growth defects of the Neo1p-depleted mutant, thus suppressing the growth defects of all the flippase mutants.

We next examined whether overexpression of *ART5* suppressed the defects in membrane trafficking. The exocytic v-SNARE Snc1p primarily localizes to the plasma membrane at polarized sites where exocytosis is occurring, such as a bud, and is recycled through the endocytic recycling pathway from the plasma membrane via early endosomes to the TGN [46]. Because the *cdc50*Δ mutant is defective in the retrieval pathway from early endosomes to the TGN [12], GFP-Snc1p accumulates intracellularly in the *cdc50*Δ *crf1*Δ mutant. However, the polarized site of GFP-Snc1p was restored to 62.0% of cells when *ART5* was overexpressed (Fig. 1C). The *neo1* mutants exhibit defects in membrane trafficking within or from the endosomal/Golgi system [21–23, 43]; accordingly, the mRFP-Snc1p in our study accumulated intracellularly in the Neo1p-depleted mutant. Overexpression of *ART5* in this mutant restored polarization of GFP-Snc1p to 62.2% of cells (Fig. 1C). These results suggest that *ART5* overexpression also suppresses the defects in membrane trafficking of the flippase mutants.

We next examined whether *ART5* overexpression restored phospholipid asymmetry in a flippase mutant. The *lem3*Δ mutant is defective in the transbilayer translocation of alkylphosphocholine drugs such as miltefosine and edelfosine as well as fluorescence-labeled PC and PE analogues across the plasma membrane. Thus, the *lem3*Δ mutant is resistant to these drugs [54], but *ART5* overexpression did not affect the miltefosine-resistance in the *lem3*Δ mutant (Fig. 1D). The *lem3*Δ mutant also exhibits growth sensitivity to cytotoxic peptides, papuamide B and duramycin, which bind to PS and PE, respectively, because these phospholipids are exposed in the exoplasmic leaflet of the plasma membrane [55, 56]. However, *ART5* overexpression did not suppress these phenotypes in the *lem3*Δ mutant (Fig. 1D). These results suggest that *ART5* overexpression suppresses flippase mutations without correction of the defects in phospholipid asymmetry.

### Mutations in the inositol transporters *ITR1* and *ITR2* suppress the growth defects of flippase mutants

**Fig 2:**
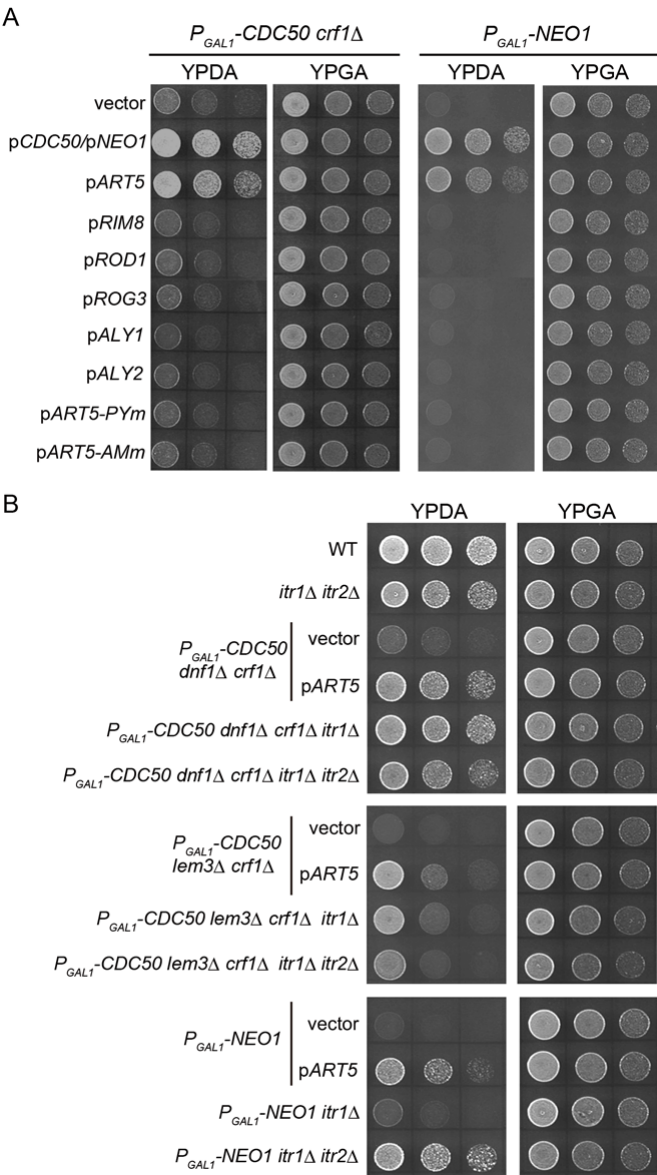
The growth defects of flippase mutants were suppressed by mutations in inositol transporters. (A) Suppression of the flippase mutations by overexpression was specific to *ART5*. Cell growth was examined as in Fig. 1A, except that the *P*_*GAL1*_ *-CDC50 crf1*Δ mutant was incubated at 25°C for 2 days. The strains used were YKT1511 (*P*_*GAL1*_ *-CDC50 crf1*Δ) and YKT1932 (*P*_*GAL1*_ *-NEO1*), both carrying YEplac195 (vector), pKT1720 (p*ART5*), pKT2135 (p*RIM8*), pKT2136 (p*ROD1*), pKT2137 (p*ROG3*), pKT1881 (p*ALY1*), pKT1882 (p*ALY2*), pKT2088 (p*ART5*-*PYm*), or pKT2138 (p*ART5*-*AMm*). YKT1511 and YKT1932 also carried pKT1263 (p*CDC50*) and pKT1469 (p*NEO1*), respectively, as positive controls. (B) Suppression of the growth defects of flippase mutants by mutations in inositol transporters. Cells were pregrown to early log phase in SGA-Ura or YPGA medium, and cell growth was examined as in Fig. 1A. The strains used were YKT1066 (wild type, WT), YKT1866 (*itr1*Δ *itr2*Δ), YKT1529 (*P*_*GAL1*_ *-CDC50 dnf1*Δ *crf1*Δ), YKT1934 (*P*_*GAL1*_ *-CDC50 dnf1*Δ *crf1*Δ *itr1*Δ), YKT1935 (*P*_*GAL1*_ *-CDC50 dnf1*Δ *crf1*Δ *itr1*Δ *itr2*Δ), YKT1513 (*P*_*GAL1*_ *-CDC50 lem3*Δ *crf1*Δ), YKT1915 (*P*_*GAL1*_ *-CDC50 lem3*Δ *crf1*Δ *itr1*Δ), YKT1914 (*P*_*GAL1*_ *-CDC50 lem3*Δ *crf1*Δ *itr1*Δ *itr2*Δ), YKT1932 (*P*_*GAL1*_ *-NEO1*), YKT1877 (*P*_*GAL1*_ *-NEO1 itr1*Δ), and YKT1881 (*P*_*GAL1*_ *-NEO1 itr1*Δ *itr2*Δ). YKT1529, YKT1513, and YKT1932 also carried YEplac195 (vector) or pKT1720 (p*ART5*).

Because there are nine arrestin homologs in budding yeast [52, 53], we next examined whether overexpression of these *ART* homologues suppressed flippase mutations. In the Cdc50p-depleted *crf1*Δ and Neo1p-depleted mutants, we overexpressed Rim8p/Art9p, Rod1p/Art4p, and Rog3p/Art7p, which are relatively similar to Art5p at the level of amino-acid sequence. Overexpression of these genes did not suppress the growth defects of the flippase mutants (Fig. 2A). We also examined Aly1p/Art6p and Aly2p/Art3p, because they are involved in early endosome-to-TGN transport, like flippases, but instead transport the general amino-acid permease Gap1p [45]. However, overexpression of neither *ALY1* nor *ALY2* suppressed the flippase mutations (Fig. 2A). Thus, the suppressor activity was specific to *ART5*.

Arrestin-related trafficking (Art) proteins are involved in the endocytic downregulation of nutrient transporters in response to excess nutrients [52, 53]. They bind to the Rsp5p HECT-domain-type ubiquitin ligase via their PY motifs, in order to ubiquitinate cargo proteins for internalization and degradation in vacuoles. When one of three PY motifs of Art5p, amino acids 532–535 (PPNY), was mutated to PANA, overexpression of the resultant mutant (*ART5-PYm*) did not suppress the flippase mutations (Fig. 2A). Art proteins contain conserved arrestin domains in the N-terminal region, which is characterized by high conservation across diverse species (the arrestin motif) [52]. We made substitutions in the arrestin motif of Art5p; overexpression of this mutant [*ART5-AMm*, Art5p (G231A/P236A/F237A)] also failed to suppress the growth defects of the flippase mutants (Fig. 2A). These results suggest that overexpression of *ART5* suppressed flippase mutations by facilitating endocytosis of a plasma-membrane transporter.

The inositol transporter Itr1p is the only identified target protein for Art5p [53]. Thus, we investigated whether the *itr1*Δ mutation suppressed the growth defects of the flippase mutants. We also examined a mutation in *ITR2*, a homologue of *ITR1*, because it encodes a minor inositol transporter [57]. As shown in Fig. 2B, *itr1*Δ, as well as *itr1*Δ *itr2*Δ mutations, suppressed growth defects in the Cdc50p-depleted *dnf1*Δ *crf1*Δ and Cdc50p-depleted *lem3*Δ *crf1*Δ mutants. The growth rate was somewhat slower in the mutants with *itr1*Δ *itr2*Δ mutations, most likely because their inositol uptake was severely compromised.

The growth defect of the Neo1p-depleted mutant was not suppressed by the *itr1*Δ single mutation, but was suppressed by the *itr1*Δ *itr2*Δ mutations. Because overexpression of *ART5* suppressed the Neo1p-depleted mutation, Art5p may also downregulate Itr2p. Of note, the Neo1p-depleted *itr1*Δ *itr2*Δ mutant grew more efficiently than the Cdc50p-depleted *lem3*Δ *crf1*Δ *itr1*Δ *itr2*Δ mutant. These results suggest that overexpression of *ART5* suppressed the growth defects of flippase mutants by endocytic downregulation of inositol transporters.

### Depletion of inositol from culture medium suppresses the defects of flippase mutants

**Fig 3:**
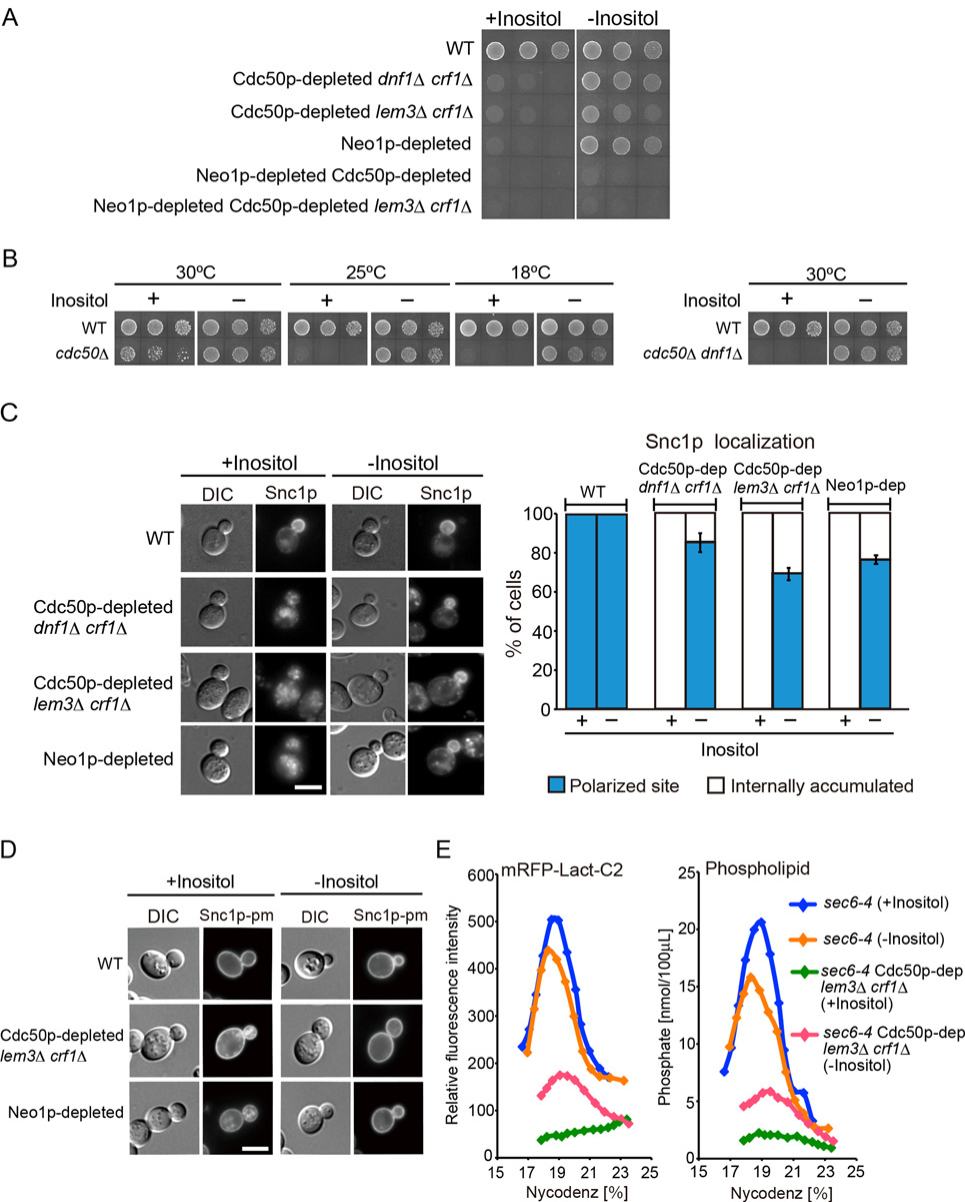
Suppression of the defects in flippase mutants by depletion of inositol from culture medium. (A) Suppression of the growth defects. Cells were pregrown to early log phase in YPGA medium, washed, and adjusted to a concentration of 1.0 × 10^7^ cells/ml. Drops of 10 μl from 5-fold serial dilutions were spotted onto an SD agar plate with or without inositol, followed by incubation at 30°C for 1.5 days. The strains used were YKT1066 (wild type, WT), YKT1529 (*P*_*GAL1*_ *-CDC50 dnf1*Δ *crf1*Δ), YKT1513 (*P*_*GAL1*_ *-CDC50 lem3*Δ *crf1*Δ), YKT1932 (*P*_*GAL1*_ *-NEO1*), YKT1909 (*P*_*GAL1*_ *-NEO1 P*_*GAL1*_ *-CDC50*), and YKT1887 (*P*_*GAL1*_ *-NEO1 P*_*GAL1*_ *-CDC50 lem3*Δ *crf1*Δ). (B) Suppression of growth defects in the flippase null mutants. Cells were pregrown to early log phase in SD medium without inositol, and cell growth was examined as in (A), except that they were incubated at 30°C, 25°C, or 18°C for 1.5, 2, or 5 days, respectively. The strains used were YKT1066 (wild type, WT), YKT1944 (*cdc50*Δ), and YKT1945 (*cdc50*Δ *dnf1*Δ). YKT1945 was constructed by tetrad dissection of spores from a *cdc50*Δ/*CDC50 DNF1*/*dnf1*Δ heterozygous diploid on an inositol-depleted SD agar plate. (C) Suppression of the defects in endocytic recycling of Snc1p. Cells were grown in SD medium with or without inositol at 30°C for 12 h or 16 h (Neo1p-depleted), followed by microscopic observation of small- or middle-budded cells. The percent of cells with mRFP-Snc1p in polarized sites was determined (n>100) and is shown with the mean ± standard deviation of three independent experiments. Representative images are shown. The strains used were YKT1912 (*mRFP-SNC1*, WT), YKT1936 (*P*_*GAL1*_ *-CDC50 dnf1*Δ *crf1*Δ *mRFP-SNC1*), YKT1937 (*P*_*GAL1*_ *-CDC50 lem3*Δ *crf1*Δ *mRFP-SNC1*), and YKT1910 (*P*_*GAL1*_ *-NEO1 mRFP-SNC1*). Bar: 5 μm. (D) Restoration of the plasma membrane location of GFP-Snc1p-pm. Cells were grown in SD-Ura or SD-Leu medium with or without inositol at 30°C for 16 h. More than 100 cells were microscopically observed, and the percent of cells with internally accumulated GFP-Snc1p-pm was determined. The strains used were YKT1066 (wild type, WT), YKT1120 (*P*_*GAL1*_ *-CDC50 lem3*Δ *crf1*Δ), and YKT1660 (*P*_*GAL1*_ *-NEO1*), carrying pKT1444 (pRS416-GFP-SNC1-pm) or pKT1491 (pRS315-GFP-SNC1-pm). Bar: 5 μm. (E) Formation of secretory vesicles. Cells were grown in SD with or without inositol at 30°C for 12 h, followed by a shift to 37°C for 2 h. Secretory vesicles were fractionated by the Nycodenz density gradient, and each fraction was measured for mRFP fluorescence intensity and the total amount of phospholipid phosphates. Strains used were YKT1844 (*sec6–4 mRFP1-Lact-C2*) and YKT1854 (*sec6–4 P*_*GAL1*_ *-CDC50 lem3*Δ *crf1*Δ *mRFP1-Lact-C2*).

We next examined whether depletion of inositol from the culture medium suppressed flippase mutations. Inositol depletion restored cell growth to an extent similar to that of the wild type in the Cdc50p-depleted *dnf1*Δ *crf1*Δ, Cdc50p-depleted *lem3*Δ *crf1*Δ, and Neo1p-depleted mutants, although the suppression was weak in the Cdc50p-depleted *lem3*Δ *crf1*Δ mutant (Fig. 3A). Inositol depletion did not suppress growth defects in the mutants simultaneously depleted for Neo1p and Cdc50p, indicating that inositol depletion does not completely bypass the essential requirement for these flippases.

To confirm that the suppression was not due to incomplete repression of the *GAL1* promoter specific to the inositol-depleted condition, we examined the *cdc*50 null mutant and found that inositol depletion suppressed its cold-sensitive growth phenotype (Fig. 3B). Furthermore, we examined whether inositol depletion suppressed the severe growth defects of the *cdc50*Δ *dnf1*Δ mutant at 30°C. To this end, we constructed this mutant by tetrad dissection on an inositol-depleted agar plate, successfully isolating the *cdc50*Δ *dnf1*Δ mutant clones. The isolated mutant did not grow on an inositol-containing plate (Fig. 3B), indicating that inositol depletion suppressed the *cdc50 dnf1* null mutations.

We also tried to isolate *cdc50*Δ *lem3*Δ *crf1*Δ and *neo1*Δ mutant clones by tetrad dissection on an inositol-depleted plate, but the *cdc50*Δ *lem3*Δ *crf1*Δ mutant grew poorly, and we failed to recover the *neo1*Δ mutant clones. These results suggest that inositol depletion does not bypass either the simultaneous loss of Cdc50p, Lem3p, and Crf1p or the loss of Neo1p. In addition, it suggests that, in the *GAL1* promoter system, cultivation in glucose-based medium cannot achieve complete depletion of Cdc50p and Neo1p.

In agreement with the suppression of growth defects, inositol depletion also suppressed the flippase mutants’ membrane trafficking defects. The polarized location of mRFP-Snc1p was restored from being in almost 0% of cells to 85.8%, 69.5%, and 76.9% of cells by the depletion of inositol in the Cdc50p-depleted *dnf1*Δ *crf1*Δ, Cdc50p-depleted *lem3*Δ *crf1*Δ, and Neo1p-depleted mutants, respectively (Fig. 3C). We recently showed that Cdc50p-depleted *lem3*Δ *crf1*Δ and Neo1p-depleted mutants also exhibit defects in the secretory pathway from the TGN to the plasma membrane [43]. Snc1p-pm, a plasma membrane—localized mutant version of Snc1p, contains point mutations that inhibit endocytosis [46]. GFP-Snc1p-pm was mislocalized to the endosomal/TGN membranes in 86.8% of the Cdc50p-depleted *lem3*Δ *crf1*Δ cells and in 87.4% of the Neo1p-depleted cells (consistent with previous results [43]), and these were decreased to 13.8% and 33.1%, respectively, by inositol depletion (Fig. 3D).

The formation of secretory vesicles can be examined by using the *sec6–4* mutation, which leads to the accumulation of secretory vesicles due to defects in vesicle fusion with the plasma membrane. We isolated secretory vesicles by Nycodenz density gradient fractionation and quantitatively examined each fraction for total phosphates (corresponding to the amount of phospholipids) and fluorescence intensity of mRFP-Lact-C2, which specifically binds to PS on the surface of secretory vesicles [43]. As shown in Fig. 3E, inositol depletion increased mRFP-Lact-C2 fluorescence intensity, as well as phospholipid concentration, in the *sec6–4* Cdc50p-depleted *lem3*Δ *crf1*Δ mutant. These results suggest that inositol depletion suppressed flippase mutations by restoring vesicle formation.

### Flippase mutations are not suppressed by either transcriptional activation of UAS_INO_-containing genes or by activation of the unfolded protein response

Inositol availability regulates the transcription of phospholipid synthesis genes that contain the inositol-responsive *cis*-acting element (UAS_INO_) [25, 33]. The Ino2p-Ino4p transcription activator complex binds to UAS_INO_, activating the transcription of genes that contain it. In the presence of inositol, Opi1p is bound to Ino2p, repressing its transcriptional activity. In the absence of inositol, phosphatidic acid (PA), a precursor of CDP-DAG, accumulates because CDP-DAG is not being consumed to synthesize PI. This accumulated PA sequesters Opi1p from the nucleus and localizes it to the ER, thereby activating the transcription of UAS_INO_-containing genes [58].

**Fig 4:**
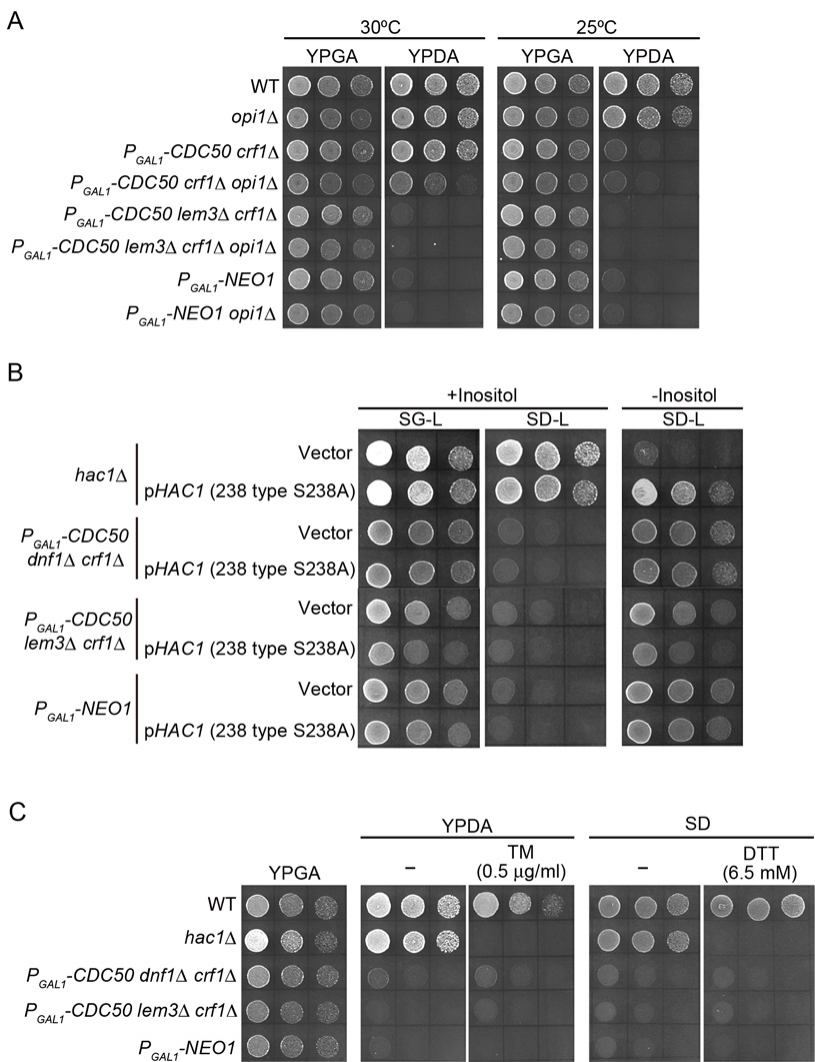
Flippase mutations are not suppressed by activation of either the Ino2p-Ino4p pathway or the unfolded protein response. (A) Activation of the Ino2p-Ino4p pathway by the *opi1*Δ mutation does not suppress the growth defects of flippase mutants. Cell growth was examined as in Fig. 1A, except the cells were incubated at 30°C or 25°C for 1 day or 2 days, respectively. The strains used were YKT38 (wild type, WT), YKT1938 (*opi1*Δ), YKT1510 (*P*_*GAL1*_ *-CDC50 crf1*Δ), YKT1939 (*P*_*GAL1*_ *-CDC50 crf1*Δ *opi1*Δ), YKT1120 (*P*_*GAL1*_ *-CDC50 lem3*Δ *crf1*Δ), YKT1940 (*P*_*GAL1*_ *-CDC50 lem3*Δ *crf1*Δ *opi1*Δ), YKT1660 (*P*_*GAL1*_ *-NEO1*), and YKT1941 (*P*_*GAL1*_ *-NEO1 opi1*Δ). (B, C) Activation of the unfolded protein response pathway (UPR) does not suppress the growth defects of flippase mutants. (B) Expression of the UPR-activating *HAC1* (238 type S238A) mutant. Cells were grown in SG-Leu medium, washed, and adjusted to a concentration of 1.0 × 10^7^ cells/ml. Drops of 10 μl from 5-fold serial dilutions were spotted onto an SG-Leu, SD-Leu, or SD-Leu without inositol agar plate, followed by incubation at 30°C for 1.5 days. The strains used were KKT466 (*hac1*Δ), YKT1529 (*P*_*GAL1*_ *-CDC50 dnf1*Δ *crf1*Δ), YKT1513 (*P*_*GAL1*_ *-CDC50 lem3*Δ *crf1*Δ), and YKT1932 (*P*_*GAL1*_ *-NEO1*), all carrying either YCplac111 (Vector) or pKT2139 [p*HAC1* (238 type S238A)]. (C) Treatment with TM or DTT. Cell growth was examined as in Fig. 1A. Cells were spotted onto a YPGA, YPDA containing 0.5 μg/ml TM, or SD containing 6.5 mM DTT agar plate, followed by incubation at 30°C for 1 day. The strains used were YKT1066 (wild type, WT), KKT466 (*hac1*Δ), YKT1529 (*P*_*GAL1*_ *-CDC50 dnf1*Δ *crf1*Δ), YKT1513 (*P*_*GAL1*_ *-CDC50 lem3*Δ *crf1*Δ), and YKT1932 (*P*_*GAL1*_ *-NEO1*).

To examine whether the derepression of UAS_INO_-containing genes is responsible for the suppression of flippase mutations under inositol-depleted conditions, the *opi1*Δ mutation was combined with flippase mutations. However, as shown in Fig. 4A, the *opi1*Δ mutation did not suppress the growth defects of the Cdc50p-depleted *crf1*Δ, Cdc50p-depleted *lem3*Δ *crf1*Δ, or Neo1p-depleted mutants. Rather, the *opi1*Δ mutation inhibited the growth of the Cdc50p-depleted *crf1*Δ mutant at 30°C. These results suggest that transcriptional activation of the UAS_INO_-containing genes does not solely account for the suppression of flippase mutations by inositol depletion.

Inositol deprivation also elicits the unfolded protein response (UPR), which is a signal transduction pathway that maintains ER homeostasis by responding to the accumulation of unfolded proteins in the ER lumen through upregulation of the expression of target genes, and a failure in the UPR results in inositol auxotrophy [33, 59, 60]. Although it remains unclear how inositol depletion triggers ER stress, many of the target genes are induced by inositol depletion [61]. Thus, using constitutive activators of UPR, we examined whether UPR activation would suppress the growth defects of flippase mutants. *HAC1* (238 type S238A) is a dominant positive mutant of the Hac1p transcription factor, which mediates transcriptional activation of the UPR target genes [47]. Expression of *HAC1* (238 type S238A), however, did not suppress the growth defects of the Cdc50p-depleted *dnf1*Δ *crf1*Δ, the Cdc50p-depleted *lem3*Δ *crf1*Δ, or the Neo1p-depleted mutants (Fig. 4B). The UPR is also induced by tunicamycin (TM) and dithiothreitol (DTT), which prevent protein glycosylation and disulfide bond formation, respectively [62]. Treatment with these drugs did not suppress the growth defects of the Cdc50p-depleted *dnf1*Δ *crf1*Δ, the Cdc50p-depleted *lem3*Δ *crf1*Δ, or the Neo1p-depleted mutant (Fig. 4C). We conclude that transcriptional activation of the UPR target genes does not solely account for the suppression of flippase mutations by inositol depletion.

### A decrease in PI level is responsible for the suppression of flippase mutations by inositol depletion

**Fig 5:**
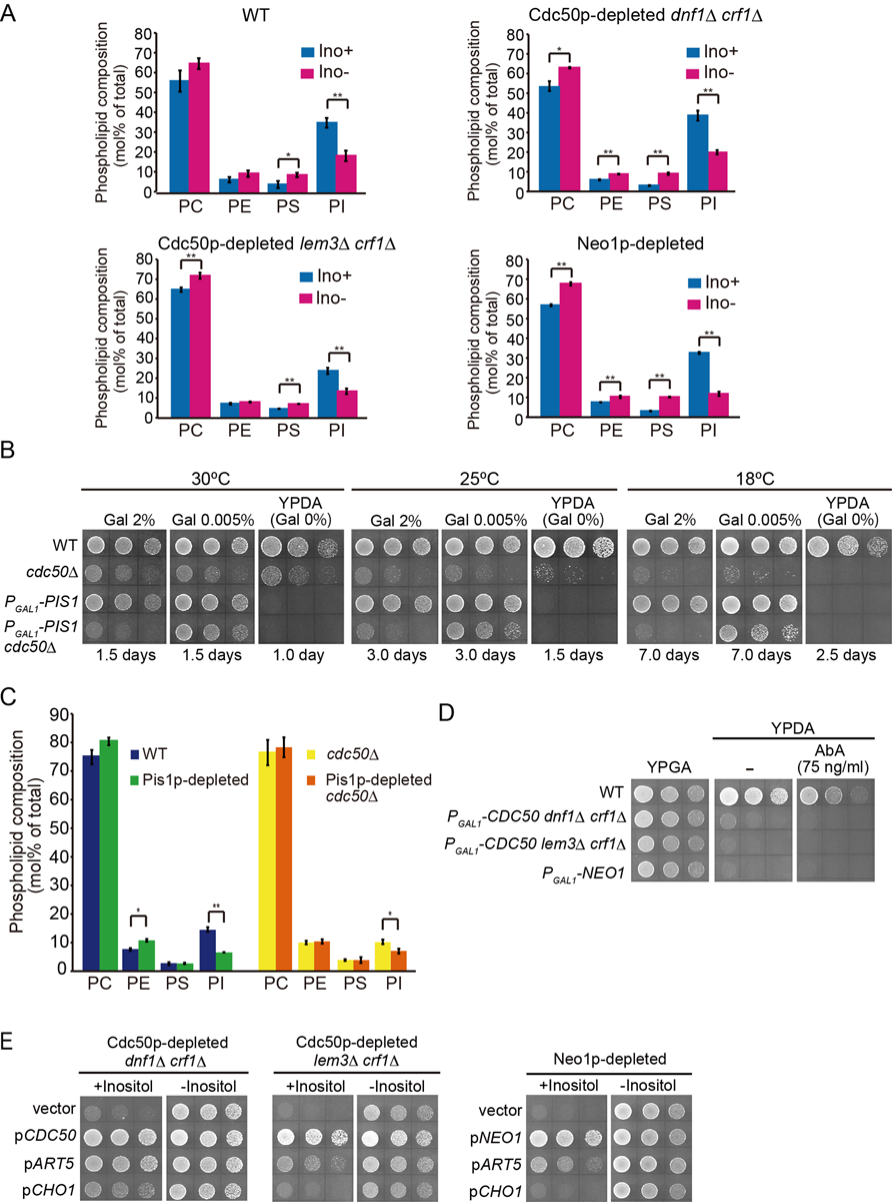
Suppression of the growth defects in flippase mutants by decreased synthesis of PI. (A) Effects of inositol depletion on steady state phospholipid composition in the flippase mutants. Cells were grown in SD medium with or without inositol at 30°C for 12 h or 16 h (Neo1p-depleted). Phospholipids were extracted and quantified by liquid chromatography mass spectrometry as described in the Materials and Methods. The data represent mole percentage (mol%) of total phospholipids, with mean values ± standard deviation (n = 3). Asterisks indicate a significant difference in the Student’s *t* test (*: *P* < 0.05; **: *P* < 0.005). The strains used were YKT1066 (wild type, WT), YKT1529 (*P*_*GAL1*_ *-CDC50 dnf1*Δ *crf1*Δ), YKT1513 (*P*_*GAL1*_ *-CDC50 lem3*Δ *crf1*Δ), and YKT1932 (*P*_*GAL1*_ *-NEO1*). (B) Suppression of the cold-sensitive growth defects in the *cdc50*Δ mutant by partial depletion of Pis1p. Cells were pregrown to early log phase in SD medium containing 2% raffinose and 2% or 0.005% galactose for 2 days, washed, and adjusted to a concentration of 1.0 × 10^7^ cells/ml. Drops of 10 μl from 5-fold serial dilutions were spotted onto the same medium and YPDA, followed by incubation at the indicated temperature for the indicated time. The strains used were YKT1066 (wild type, WT), YKT1697 (*cdc50*Δ), YKT1942 (*P*_*GAL1*_ *-PIS1*), and YKT1943 (*P*_*GAL1*_ *-PIS1 cdc50*Δ). (C) Effects of Pis1p depletion on steady state phospholipid composition in the *cdc50*Δ mutant. Cells were pregrown and grown in SD medium containing 2% raffinose and 0.005% galactose for 4 days, in total, at 30°C. Phospholipids were extracted and quantified as in (A). The strains used were those in (B). (D) Aureobasidin A (AbA) treatment does not suppress the growth defects of flippase mutants. Cell growth was examined as in Fig. 1A. Cells were spotted onto a YPGA, YPDA, or YPDA containing 75 ng/ml AbA agar plate, followed by incubation at 30°C for 1 day. The strains used were YKT1066 (wild type, WT), YKT1529 (*P*_*GAL1*_ *-CDC50 dnf1*Δ *crf1*Δ), YKT1513 (*P*_*GAL1*_ *-CDC50 lem3*Δ *crf1*Δ), and YKT1932 (*P*_*GAL1*_ *-NEO1*). (E) *ART5* overexpression suppressed the growth defects of flippase mutants more efficiently than the *CHO1* overexpression. Cells were pregrown to early log phase in SG-Leu medium, washed, and adjusted to a concentration of 2.5 × 10^7^ cells/ml. Drops of 10 μl from 5-fold serial dilutions were spotted onto an SDA agar plate with or without inositol, followed by incubation at 30°C for 1 day or 1.5 days (the *P*_*GAL1*_ *-CDC50 lem3*Δ *crf1*Δ mutant). The strains used were YKT1529 (*P*_*GAL1*_ *-CDC50 dnf1*Δ *crf1*Δ), YKT1513 (*P*_*GAL1*_ *-CDC50 lem3*Δ *crf1*Δ), and YKT1932 (*P*_*GAL1*_ *-NEO1*), carrying YEplac181 (vector), pKT1259 (p*CDC50*), pKT1788 (p*NEO1*), pKT1719 (p*ART5*), or pKT1754 (p*CHO1*).

Inositol depletion not only decreases the rate of PI synthesis, but it also increases the rate of PS synthesis by diverting CDP-DAG to PS synthesis [63, 64]. This increase results in elevated synthesis of PE and PC, because PS is converted to PE, which is subsequently converted to PC. Because of the effects of inositol on phospholipid synthesis, we examined the effect of inositol depletion on the steady-state phospholipid composition of the flippase mutants using liquid chromatography mass spectrometry. In inositol-depleted wild-type cells, PI was significantly decreased from 34.7 ± 2.4% of the total phospholipids to 18.0 ± 2.6% (Fig. 5A). By contrast, the levels of the other three phospholipids were slightly increased, as expected. In the Cdc50p-depleted *dnf1*Δ *crf1*Δ mutant, phospholipid composition was very similar to that of the wild type, both in the presence and absence of inositol. In the Cdc50p-depleted *lem3*Δ *crf1*Δ mutant, the PI level was lower (23.7 ± 1.6% of phospholipids) than that of the wild type in the presence of inositol, but significantly decreased to 13.5 ± 1.4% after inositol depletion. In the Neo1p-depleted mutant, the extents of both the PI decrease and PS increase were higher than that of wild type: the PI level decreased from 32.6 ± 0.5% to 11.8 ± 1.0% of phospholipids, and the PS level increased from 2.9 ± 0.0% to 10.2 ± 0.2%. In general, the flippase mutants mirrored the wild-type cells in both conditions, with the PI level being lower under inositol-depleted conditions, and the PS, PE, and PC levels being mildly higher.

We next examined whether a reduction in PI synthesis would suppress a flippase mutation. Cellular PI level can be attenuated by regulating expression of the phosphatidylinositol synthase gene *PIS1* [65]. In this study, *PIS1* was expressed under the control of the *GAL1* promoter, with low concentrations of galactose in the raffinose-based medium. Because *PIS1* is an essential gene, full repression of *PIS1* transcription in glucose-based medium (YPDA) would result in severe growth defects. In our study, attenuated expression of *PIS1* at 0.005% galactose suppressed the cold-sensitive growth defects of the *cdc50*Δ mutant (Fig. 5B). We next examined the phospholipid composition of cells that were grown in the same condition (0.005% galactose medium at 25°C). In the wild-type background, attenuated expression of *PIS1* significantly decreased PI level from 14.0 ± 0.8% to 4.9 ± 0.1% of phospholipids. In the *cdc50*Δ background, the PI level was lower (10.8 ± 0.8%) than in the wild type, but attenuated expression of *PIS1* decreased it further to 7.2 ± 0.9%. By contrast, we did not observe any significant changes in PS level after *PIS1* expression was attenuated (Fig. 5C). Therefore, a decrease in the PI level may be responsible for the suppression of the *cdc50*Δ mutation.

PI serves as a precursor for signaling molecules and lipids, including sphingolipids and phosphoinositides. Thus, inhibiting the synthesis of these molecules might suppress the flippase mutations. Treatment with aureobasidin A (AbA), a specific inhibitor of the inositol phosphorylceramide synthase Aur1p, reduces complex sphingolipids levels [66]. However, AbA treatment did not suppress the growth defects of the Cdc50p-depleted *dnf1*Δ *crf1*Δ, the Cdc50p-depleted *lem3*Δ *crf1*Δ, or the Neo1p-depleted mutant (Fig. 5D). These results suggest that a reduction in the complex sphingolipid level does not solely account for the suppression of flippase mutations by inositol depletion. We did not examine the effects of reducing phosphoinositides, because phosphatidylinositol-4-phosphate is rather required for Drs2p function [67, 68].

We showed previously that overexpression of the phosphatidylserine synthase gene *CHO1* partially suppresses flippase mutations, most likely by increasing the synthesis of PS [23]. Thus, a slight increase in PS caused by inositol depletion could also be responsible for the suppression of flippase mutations. We compared the suppressor activity of overexpressed *CHO1* and *ART5* in numerous flippase mutants (Fig. 5E). Overexpression of *CHO1* weakly suppressed the growth defects of the Cdc50p-depleted *dnf1*Δ *crf1*Δ mutant, but not those of the Cdc50p-depleted *lem3*Δ *crf1*Δ or Neo1p-depleted mutants. By contrast, overexpression of *ART5* suppressed all the flippase mutations, and inositol depletion suppressed them to an extent comparable to that of wild type. These results are consistent with our hypothesis that a decrease in PI level may be responsible for the suppression of flippase mutations by inositol depletion, although we cannot exclude the possibility that an increase in PS is also involved in the suppression.

## Discussion

Numerous gene-deletion mutants require inositol for growth [69, 70], presumably because (i) inositol is an essential precursor for the biosynthesis of PI and PI-derived molecules, (ii) inositol is involved in transcriptional regulation of numerous genes, and (iii) inositol depletion is a stress-inducing growth condition. Thus, it was surprising that the essential requirement for flippases was partially bypassed by inositol depletion. Investigating these suppression mechanisms could be important for determining why eukaryotic cells have evolved phospholipid flippases.

Inositol depletion caused prominent changes in membrane lipid composition, including a decrease in PI. Our results suggest that inositol depletion restores vesicle formation from the endosomal membranes, perhaps by changing the lipid composition of the endosomal membranes without correction of perturbed phospholipid asymmetry to partially bypass the requirement for flippases in vesicle formation. We previously found that overexpressing *CHO1*, which encodes phosphatidylserine synthase, weakly suppresses flippase mutations, most likely due to increased levels of PS [23]. In inositol-depleted cells, the PS level was somewhat higher than that in cells grown in the presence of inositol. Thus, an increase in PS may also contribute to suppression. However, suppression by inositol depletion was much more efficient than suppression by *CHO1* overexpression. Because the most prominent change caused by inositol depletion was a decrease in PI, we hypothesize that this change is primarily responsible for suppression. Consistent with this, depletion of PI, caused by partial repression of *PIS1* expression, suppressed the *cdc50*Δ mutation without a significant increase in PS.

Flippases are involved in vesicle formation from the plasma membrane during the endocytosis pathway [71], from the TGN during the secretory pathway [17], and from early endosomes during the endocytic recycling pathway [12, 72]. Phospholipid flipping by flippases has been implicated in inducing a local membrane curvature that would assist in vesicle formation [24]. A crucial factor that can suppress this curve formation is membrane rigidity. The plasma membrane is rich in sterols and sphingolipids [73–75]. In addition, phospholipids are more saturated in the plasma membrane than in the ER [76]. Preferential interaction of sterols with the saturated acyl chains of phospholipids and sphingolipids results in tight lipid packing, making the plasma membrane a rigid and thick barrier between intracellular and extracellular spaces [77]. Thus, flippases may have evolved to form a vesicle by counteracting the high rigidity of the plasma membrane and its related endosomal/TGN membranes. A decrease in membrane rigidity may reduce dependency on flippases for curvature formation, and thus may restore vesicle formation in flippase mutants. Of note, acyl chains are more saturated in PI than in other phospholipids [76]. Thus, inositol depletion may suppress flippase mutations by decreasing the membrane rigidity of endosomal/TGN membranes.

Membrane rigidity increases with the fraction of charged lipid species: stronger repulsion between the polar heads of charged lipids effectively suppresses membrane undulations [78]. Thus, a decrease in charged species may reduce the requirement for flippases in vesicle formation by decreasing membrane rigidity. PI and PS are major species of charged phospholipids. Although the net membrane charge is maintained at a constant level under various growth conditions [79], a lower local concentration of PI (e.g. endosomal membranes) might contribute to vesicle formation in the absence of flippases by reducing membrane rigidity.

Transcriptional analyses suggested that inositol regulates the transcription of numerous genes [61, 80, 81], but we did not find any gene that was related to the vesicular transport functions of flippases (such as clathrin adaptors). Depletion of inositol causes changes in lipid metabolism, not only because it is a precursor of PI, but because it also transcriptionally activates the UAS_INO_-containing genes that are involved in phospholipid biosynthesis (*INO1*, *OPI3*, *PSD1*, *CHO1*, *CDS1*, *CKI1*, etc.). Inositol depletion also transcriptionally activates UPR target genes, as well as many other genes, including those with unknown functions that are not under the control of either UAS_INO_ or UPR. Although forced transcriptional activation of the UAS_INO_-containing genes or the UPR pathway did not suppress flippase mutations, it is possible that combinations of these responses contributed to the suppression observed during inositol depletion. Furthermore, inositol depletion activates the PKC-MAPK pathway, albeit transiently [82, 83], and could affect the synthesis of phosphoinositides [27], inositol pyrophosphates [28, 29], and GPI anchor proteins [32] that have PI as a precursor, all of which have not been examined in this study. Phosphatidylinositol-4-phosphate is rather implicated in the activation of Drs2p flippase activity [68], but a decrease of other phosphoinositides might be involved in the suppression of flippase mutations. The functional relationships between the other responses, or the effects they produce, and vesicle formation remain unclear, but they might be indirectly or partially involved in the suppression as well.
